# A Ball Valve Thrombus in a Young Obese Male With Hyperlipidemia

**DOI:** 10.7759/cureus.44625

**Published:** 2023-09-04

**Authors:** Dibyasundar Mahanta, Debasish Das, Debasis Acharya

**Affiliations:** 1 Cardiology, All India Institute of Medical Sciences, Bhubaneswar, Bhubaneswar, IND

**Keywords:** hyperlipidemia, clot, dyslipidemia, obesity, thrombus, valve, ball

## Abstract

A ball valve thrombus is a common entity in rheumatic mitral stenosis where a large clot forms in the left atrial appendage, the clot detaches from the left atrial appendage, swirls in the left atrium in a clockwise manner, obstructs the mitral inflow producing cyclic acute pulmonary edema, and likely causes left ventricular outflow tract obstruction producing transient syncope or embolism to the brain producing dense hemiplegia. Besides rheumatic mitral stenosis, ball valve thrombus has been described in post-mitral valve replacement patients and restrictive cardiomyopathy. Left atrial myxoma sometimes rocks across the mitral valve producing the effect of a ball valve thrombus. Right atrial ball valve thrombus has been described after prolonged parenteral nutrition through a central line in intensive care units and in carcinoma patients with cardiac metastasis. We report an extremely rare case of a ball valve thrombus in an obese individual where dyslipidemia was the sole triggering factor for the formation of a ball valve thrombus in the left atrium.

## Introduction

In critical mitral stenosis, due to mitral inflow obstruction, blood flow velocity across the orifice of the left atrial appendage slows down to less than 40 cm/second and blood starts to clot inside the left atrial appendage. Hence, the left atrial appendage becomes a nidus for thrombus formation in critical mitral stenosis. Sometimes a large thrombus forms inside the left atrial appendage, which detaches from the wall of the left atrial appendage, swirls inside the left atrium in a clockwise manner, and obstructs the mitral valve producing acute pulmonary edema known as a ball valve thrombus. This freely moving large clot inside the left atrium is prone to produce massive hemispheric embolization and catastrophic stroke in middle-aged and elderly individuals. Paroxysmal atrial fibrillation in these patients also makes them prone to developing thrombus inside the left atrial appendage, as during atrial fibrillation and fast ventricular rate, the left atrium behaves as a lake where the blood pool remains relatively stagnant producing a thrombus inside the atrium. The most important aspect of a ball valve thrombus is that sometimes it produces acute left ventricular outflow tract obstruction, hemodynamic collapse, and syncope before producing a hemispheric stroke. Post-cardiac surgery, especially post-mitral valve surgery, postoperative coagulopathy besides mechanical mitral valve in position also predisposes to the formation of a ball valve thrombus in the left atrium [1]. The huge left atrium and persistent atrial fibrillation in restrictive cardiomyopathy also predispose individuals to the development of a ball valve thrombus inside the left atrium [2]. Right atrial ball valve thrombus is a rare phenomenon. Cardiac metastasis from the Wilms tumor sometimes forms a ball valve thrombus inside the right atrium where the thrombus extension from the inferior vena cava detaches and forms a ball valve thrombus inside the right atrium. Prolonged hyperalimentation produces a right atrial ball valve thrombus where a right atrial central line acts as a foreign nidus for the formation of a thrombus inside the right atrium [3]. Here, we report an extremely rare case of left atrial ball valve thrombus where obesity and dyslipidemia were the sole triggering factors for the formation of a ball valve thrombus inside the left atrium without any history of paroxysmal atrial fibrillation. Obesity and dyslipidemia can also trigger a malignant clot inside the left atrium producing a ball valve thrombus.

## Case presentation

**Figure 1:**
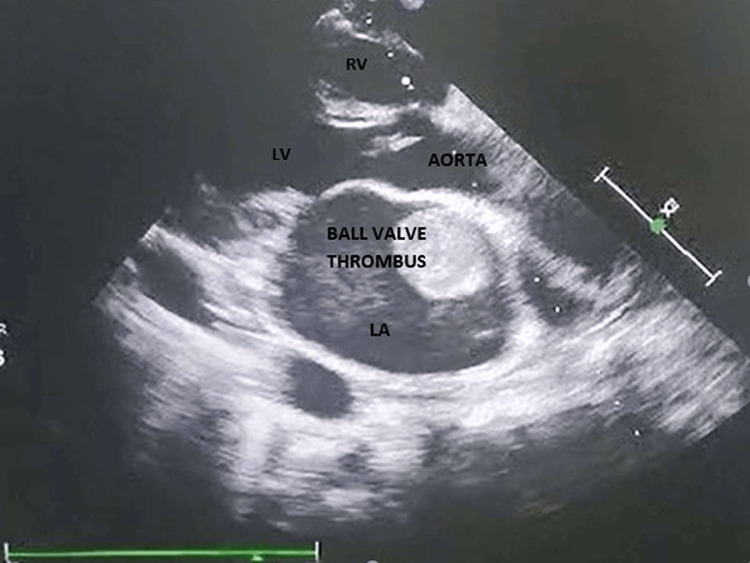
Highly mobile thrombus ball in the left atrium. LA: left atrium; LV: left ventricle; RV: right ventricle

**Figure 2:**
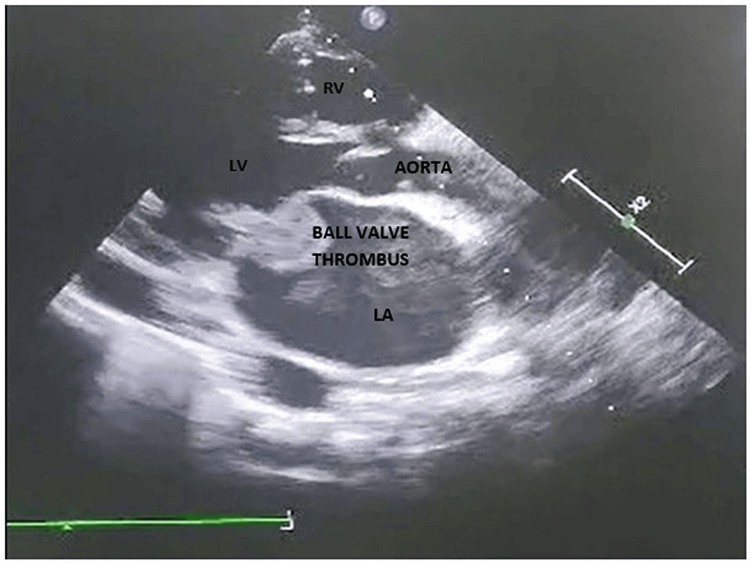
Ball valve thrombus across the mitral valve in obesity. LA: left atrium; LV: left ventricle; RV: right ventricle

**Figure 3:**
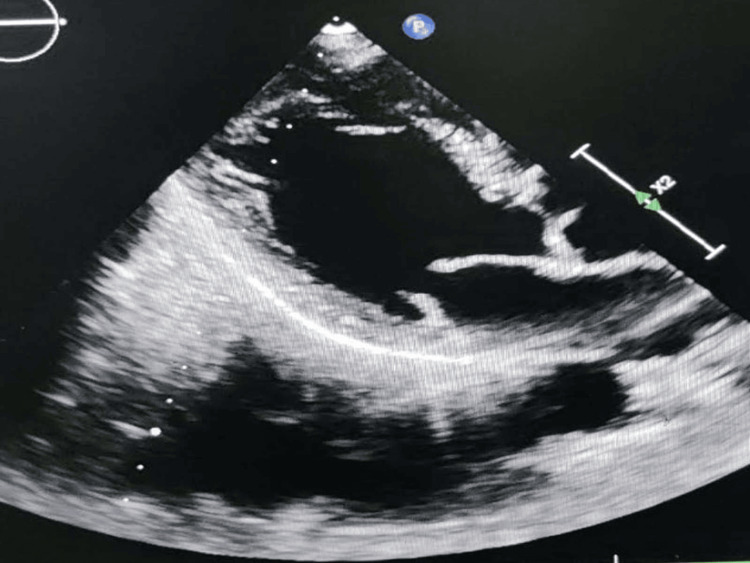
Parasternal long-axis view showing complete resolution of the ball valve thrombus with anticoagulation.

**Figure 4:**
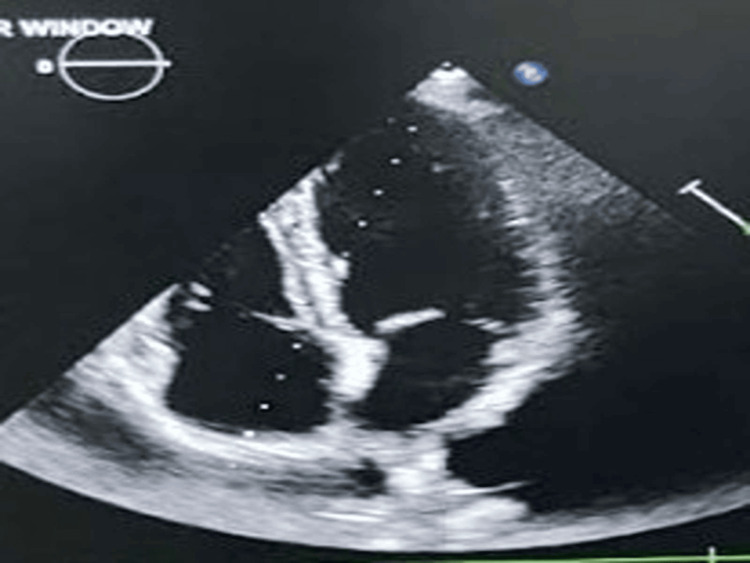
Apical four-chamber view showing complete resolution of the ball valve thrombus with anticoagulation.

A 26-year-old male presented to the emergency department with a history of acute-onset shortness of breath with orthopnea and paroxysmal nocturnal dyspnea for the last two days without any chest pain, palpitation, presyncope, or syncope. He was a nonalcoholic and nonsmoker without any family history of coronary artery disease. He did not reveal any history of acute rheumatic fever in early childhood and was not on intramuscular benzathine penicillin prophylaxis. He had morbid obesity with a body mass index of 37 kg/m^2^. All serum chemistries were within normal limits except for dyslipidemia with a low-density lipoprotein (LDL) level of 197 mg/dL, total cholesterol of 264 mg/dL, and triglyceride (TG) of 335 mg/dL. A chest X-ray posteroanterior view revealed cephalization suggestive of passive pulmonary venous congestion. His N-terminal pro-brain natriuretic peptide level was 366 ng/mL suggestive of acute heart failure. Bedside echocardiography was done to evaluate the cause of dyspnea which revealed a freely mobile thrombus of 15 mm × 15 mm (Figures 1, 2) intermittently occluding the mitral inflow producing a ball valve effect. Thorough history taking revealed a history of transient ischemic attack with peripheral tingling and numbness and right hemiparesis seven days back which had resolved on its own within 24 hours. CT brain imaging following the episode was normal. He had a normal bilateral carotid Doppler study with normal carotid intimal medial thickness. An ultrasound of the abdomen and pelvis also revealed no mass lesion, and there was no thrombus in the inferior vena cava which might have detached forming a feely mobile thrombus in the left atrium. Our case is a unique demonstration of a freely floating highly mobile left atrial thrombus in an obese patient with dyslipidemia. The patient’s plasma homocysteine, Lp (a), and Factor V Leiden were within normal limits. His protein C and protein S were also within normal limits. Contrast-enhanced CT of the thorax and abdomen also ruled out the presence of any occult malignancy. The patient was treated with intravenous unfractionated heparin 5,000 IU six hourly with overlapping warfarin 2 mg/day for 10 days. The floating mass in the left atrium resolved after eight days of extended unfractionated heparin with oral anticoagulation (Figures 3, 4). Prothrombin time (PT)/international normalized ratio (INR) was maintained between 2 and 3 during the therapeutic regimen with anticoagulants. He had no more episodes of transient ischemic attack during the hospital stay. The most important catastrophe with a ball valve thrombus is that they sometimes embolize causing large hemispheric stroke with dense hemiplegia. Dyslipidemia itself damages the endothelium with oxidized LDL and makes the nude endothelium prone to thrombus. Although a rough McCallum’s patch in the posterior surface of the left atrium can be thrombogenic in rheumatic mitral stenosis, obesity with dyslipidemia can itself form a thrombotic milieu inside the heart, as demonstrated in our young patient having a large left atrial ball valve thrombus. Besides rheumatic and degenerative mitral valve disease, prosthetic mitral valve in situ, and restrictive cardiomyopathy, dyslipidemia in obesity can also be a trigger for forming a large systemic freely floating mobile thrombus in a relatively young adult.

## Discussion

Left atrial smoke is the precursor for the formation of left atrial thrombus in patients with mitral stenosis. In critical rheumatic stenosis, a clot initially forms inside the left atrial appendage, detaches itself from the left atrial appendage wall, starts to swirl inside the left atrium, and intermittently pops out of the mitral valve forming a ball valve thrombus. A ball valve thrombus physiologically behaves like critical mitral stenosis with an acute rise in left atrial pressure secondary to severe mitral inflow obstruction, passive pulmonary venous congestion, pulmonary edema with subsequent paroxysmal nocturnal dyspnea, and orthopnea, as noted in our patient. Besides rheumatic mitral valve disease, ball valve thrombus has been noted in degenerative mitral valve disease [4], restrictive cardiomyopathy, and post-cardiac surgery, especially prosthetic mitral valve in situ. Right atrial ball valve thrombus is a rare event to be reported in prolonged parenteral nutrition with an intrajugular central line in situ. Two notable complications with ball valve thrombus are embolic large dense hemispheric stroke and syncope secondary to acute left ventricular outflow tract obstruction. Adequate systemic anticoagulation with maintenance of PT/INR between 2 and 3 prevents the formation of ball valve thrombus in critical mitral stenosis. Sometimes large left atrial myxoma exhibits a wrecking motion across the mitral valve and behaves like a ball valve thrombus [5]. Although calcified right atrial ball valve thrombus has been reported in morbid obesity [6], left atrial ball valve thrombus has not been reported so far. The described case of isolated right atrial ball valve thrombus had severe pulmonary artery hypertension with right ventricular diastolic failure which was the predisposing factor for the formation of right atrial ball valve thrombus [6]. Dyslipidemia in obesity produces endothelial dysfunction [7] which predisposes to thrombosis in the left atrium or left atrial appendage and may be the plausible cause for the formation of such a large malignant clot inside the left atrium. The presence of a ball valve thrombus in morbid obesity has not yet been described in the literature. Besides dyslipidemia producing endothelial dysfunction, oxidized LDL in dyslipidemia also acts as a predisposing factor for vascular or chamber thrombosis. The patient was strictly monitored for neurological status with adequate systemic heparinization and anticoagulation with warfarin which dissolved the malignant clot in eight days. Extended heparin therapy (for more than five days) in some cases helps in achieving successful clot lysis, prevention of stroke, and achieving a good clinical outcome. As the patient had a history of recent transient ischemic attack, we did not perform thrombolysis for the patient with streptokinase, reteplase, or tenecteplase. Although left atrial ball valve thrombus is a rare clinical entity, adequate systemic anticoagulation and neuromonitoring can save the patient from a catastrophic large hemispheric stroke, especially in young patients.

## Conclusions

Our case is an interesting and extremely rare illustration of a ball valve thrombus in an obese individual where dyslipidemia with a high LDL level (>190 mg/dL) was the provoking factor for the formation of a large clot inside the left atrium. Besides rheumatic mitral stenosis, degenerative calcific mitral valve disease, restrictive cardiomyopathy, and post-cardiac surgery hypercoagulable state with a prosthetic mitral valve in situ, isolated obesity with dyslipidemia can also be a predisposing factor for the formation of a ball valve thrombus in the left atrium. Early recognition of such ball valve thrombi with adequate systemic heparinization with anticoagulation can save young patients from a catastrophic large embolic hemispheric stroke.
